# Transdermal Delivery of Sonidegib via Iontophoresis from PEDOT:PSS/Gelatin Hydrogels for Basal Cell Carcinoma Skin Cancer

**DOI:** 10.3390/pharmaceutics18040494

**Published:** 2026-04-17

**Authors:** Phimchanok Sakunpongpitiporn, Johannes Schwank, Napa Parinyanitikul, Sutima Luangdilok, Nattaya Teeyapun, Aumpika Kesornsit, Anuvat Sirivat

**Affiliations:** 1 The Petroleum and Petrochemical College, Chulalongkorn University, Bangkok 10330, Thailand; phimchanok.s18@gmail.com; 2 Department of Chemical Engineering, University of Michigan, Ann Arbor, MI 48109, USA; schwank@engin.umich.edu; 3 Division of Medical Oncology, Department of Medicine, Faculty of Medicine, Chulalongkorn University, Bangkok 10330, Thailand; napaparinyanitikul@gmail.com (N.P.); nattaya.te@chula.ac.th (N.T.); 4 The King Chulalongkorn Memorial Hospital, Bangkok 10330, Thailand; 5 Department of Biochemistry, Faculty of Medicine, Chulalongkorn University, Bangkok 10330, Thailand; sutima.l@chula.ac.th; 6 Faculty of Pharmaceutical Sciences, Burapha University, Chonburi 20131, Thailand; aumpika.ke@go.buu.ac.th

**Keywords:** PEDOT:PSS, gelatin, hydrogel, iontophoresis, basal cell carcinoma, cancer drug

## Abstract

**Background/Objectives**: Skin cancers belong to the most frequent cancer type with over a million cases per year. Presently, transdermal drug delivery systems (TDDS) are an attractive drug delivery route, but they still face some limitations due to the resistance of human skin. **Methods**: Here, Sonidegib, PEDOT:PSS, and gelatins were employed as the model drug, drug carrier, and drug matrix, respectively. **Results**: Gelatin hydrogels were fabricated via the physical crosslinking to avoid toxicity towards the human skin. PEDOT:PSS was synthesized by chemical oxidative polymerization as the drug carrier. Sonidegib first interacted with PEDOT:PSS before they were embedded into the gelatin hydrogels. In the release and release-permeation experiments, the amounts of Sonidegib released and permeated were investigated under the effects of gelatin types, concentrations, pH values, PEDOT:PSS, and electrical voltages. For the effect of gelatin types, the BG gelatin provided higher amounts of Sonidegib release than PG from the higher electrorepulsive force. Under applied electrical voltages and with PEDOT:PSS present, the amounts of Sonidegib release and release-permeation amounts increased as PEDOT:PSS assisted in providing higher electroosmosis and electrorepulsive forces. **Conclusions**: In summary, PEDOT:PSS in the BG hydrogel is demonstrated here as a potential drug carrier to improve the Sonidegib release and release-permeation iontophoretically for TDDS.

## 1. Introduction

Cancer occurs when abnormal cells grow, which may spread to other body parts [1]. The World Health Organization (WHO) reported in 2018 that cancer deaths were 9.6 million, or one in six deaths among the overall 57.6 million cancer patients. As of 2022, there were 20 million and 9.7 million new cancer cases and deaths reported, respectively [2]. It is anticipated that in 2040, new cancer cases per year will reach 29.9 million, with the death rate per year at 15.3 million. The National Cancer Institute revealed that there are more than 100 cancer types, with skin cancer being a common type with over one million new cases per year [3]. In 2020, WHO indicated that skin cancer was the world’s fifth most common cancer amongst all cancers [4].

Skin cancer can be classified into three types, namely the basal cell carcinomas (BCC), squamous cell carcinomas (SCC), and melanoma (CM) [5]. BCC is the most common skin cancer, accounting for 75% of all skin cancers. WHO predicted that the number of BCC patients increased by 6.8% per year due to increasing exposures to the ultraviolet radiation in sunlight and artificial light sources, recreational activities, and depletion of the ozone layer [6]. BCC can appear anywhere on the body, but it usually appears on the face and neck. BCC grows slowly and rarely metastasizes, but it can cause local tissue destruction and disfigurement [7]. Presently, there are various therapeutics to treat BCC, including surgery and radiation therapy, systemic chemotherapy, and topical pharmacological treatments. The methods of treatment depend on many factors, such as tumor location, size, and the patient’s overall health. The most common treatment is surgery, but many patients are quite concerned with its aesthetic impact on visible areas, especially the face. On the other hand, radiation therapy possesses disadvantages such as high-frequency treatment and side effects such as dermatitis, skin thinning, and hair loss [8].

The Food and Drug Administration (FDA) have approved several drugs to treat BCC, including Imiquimod, 5-fluorouracil (5-FU), Diclofenac, Ingenol Mebutate, Tirbanibulin, Vismodegib, and Sonidegib [9]. Sonidegib or Erismodegib is an antineoplastic agent used for the treatment of locally advanced or recurrent BCC following surgery and radiation therapy, and for the BCC patients who wish to avoid surgery or radiation therapy. It is classified as a targeted cancer drug that acts as an inhibitor of the hedgehog (Hh) signaling pathway. This drug binds to the transmembrane proteins that are involved with the Hh signal transduction, resulting in the obstruction of tumor cell proliferation, growth, and ultimate death [6].This drug is usually administered through an oral route, but the disadvantages of this route are the first pass metabolism, uncontrollable drug adsorption, and unusable with unconscious patients [10]. Therefore, TDDS are a possible route to deliver BCC drugs directly into the skin.

TDDS are a painless method of self-administration. The advantages of TDDS are avoiding the first-pass effect, with a controllable drug rate, amount, and duration, a prolonged and steady state drug profile, and a uniform plasma level [11]. The applicable drug properties are low molecular weight, balanced lipophilicity, and measurable solubility both in oil and water [12]. However, the adsorption in the skin occurs as the drugs must pass through the stratum corneum acting as the barrier. To overcome this limitation, many methods can be applied in TDDS, such as chemical enhancers, sonophoresis, microneedles, electroporation, and iontophoresis [13]. Iontophoresis employs a low electrical current (0.1–1.0 mA/cm^2^) without endangering human skin, to push the drug through and to reduce the skin resistance [14]. Specifically, this technique can manipulate the drug rate, amount, and duration [15].

One kind of matrix to accommodate drugs is hydrogels due to their excellent biocompatibility, biodegradability, and cytocompatibility [16]. Hydrogels are three-dimensional networks or interconnected porous structures which can absorb and retain water in their porous structures. Hydrogels can be fabricated via physical or chemical crosslinking, made up of entangled polymer chains, hydrogen bonds, and covalent chemical bonds [17]. For medical purposes, physical crosslinking is more preferable than chemical crosslinking due to its non-toxicity, higher water absorption, and a reversible network. Physically crosslinked hydrogels are readily fabricated from several bio-based polymers such as agarose, silk fibroin, carragenan, gelatin, and agar [18].

Gelatin is a natural biopolymer from collagen in the skin, bones, and connective tissues [19]. Gelatin is commonly used in tissue engineering and biomedical applications because of its excellent properties such as biocompatibility, biodegradability, renewability, cost-effectiveness, nontoxicity, and thermal reversibility [20]. Gelatin hydrogels can be formed via physical or chemical crosslinking. Physical crosslinking is through the conformational changes from an arbitrary coil to a triple helix via changes in pH, temperature, ionic interactions, and hydrogen bonding [21]. Physical crosslinking is preferable in TDDS due to concerns about human skin from toxic chemical crosslinkers.

However, the limitations of using hydrogels in TDDS are the slow drug release rate and the inability to deliver large drug molecules. However, the TDDS from a hydrogel can be improved by iontophoresis and even further by adding a conductive polymer as a drug carrier [22]. Conductive polymers (CPs) are organic polymers possessing high electrical conductivity, as electrons can delocalize through their conjugated systems [23]. CPs are promising drug carriers because they can respond to electrical stimuli to control the drug release, in addition to improving the electrical conductivity of the matrix. Examples of CPs are Poly(3,4-ethylenedioxythiophene) (PEDOT), Polypyrrole (PPY), and Polyaniline (PANI). The combination of hydrogels and CPs creates interpenetrating networks with the higher hydrogel swelling and mesh size, leading to improved drug release [24]. Poly(3,4-ethylenedioxythiophene):polystyrene sulfonate (PEDOT:PSS) is one of the CPs chosen here because of its excellent properties, namely high electrochemical stability, high electrical conductivity, high biocompatibility, high solubility in water, and the ease of forming the hydrogel by itself [25].

In previous works, Gamal et al. (2021) [26] investigated sonidegib-loaded ethosomes and liposomes to treat skin cancer. In the in vitro drug release, the amounts of sonidegib released from ethosomes and liposomes were 61.87 ± 0.57% and 45.83 ± 0.62%, respectively. The corresponding amounts of sonidegib release permeation were 154.74 ± 1.72 μg/cm^2^ and 77.35 ± 0.89 μg/cm^2^, respectively. Pedersen et al. (2022) [27] studied sonidegib release via topical delivery. In the preclinical studies, porcine skin was used; the concentrations and times of sonidegib release were 1–1.5 μg/g porcine skin and 1–8 h, respectively. The murine hair regrowth in the mouse model was treated once daily, and hair growth was inhibited within 15 days. Currently, there are no research works which have reported the use of the iontophoresis technique to deliver sonidegib release.

In this work, a gelatin hydrogel was used as the matrix, as it can be physically crosslinked via heat without using a crosslinking agent. It provided non-toxicity and high water absorption suitable for transdermal drug delivery. Iontophoresis was then utilized to manipulate the drug release rate, amount, and duration, as this technique provided a pushing force on the drug through the skin, and at the same time reduced the skin resistance. In addition, PEDOT:PSS was also used as the drug carrier to further enhance sonidegib release due to its high electrical conductivity and high solubility in water. The effects of gelatin types, concentrations, pH values, the presence of PEDOT:PSS as the drug carrier, and electric field strengths on the drug release rate, amount, and duration from the gelatin hydrogels were systematically investigated and will be presented here.

## 2. Materials and Methods

### 2.1. Materials

Bovine skin gelatin (BG) (Sigma Aldrich, Darmstadt, Germany) and Porcine skin gelatin (PG) (Sigma Aldrich) were used as the hydrogel materials. Sonidegib (Combi Blocks, San Diego, CA, USA) was used as the skin cancer model drug. 3,4-Ethylenedioxythiophene (EDOT) (Sigma Aldrich), poly(styrenesulfonate) (PSS) (M_w_ = 75,000 gmol^−1^; Sigma Aldrich), and sodium persulfate (SP) (Sigma Aldrich) were used for synthesizing PEDOT:PSS. Methanol and acetone (RCI Labscan) were used for washing the PEDOT:PSS precipitate. Sodium phosphate monobasic dihydrate (SPMD) (Sigma Aldrich) and sodium phosphate dibasic dihydrate (SPDD) (Sigma Aldrich) were used for preparing the PBS buffers. Deionized water (DI water) and ethanol (RCI Labscan, Bangkok, Thailand) were utilized as solvents. The CRL-2522 (American Type Culture Collection, Manassas, VA, USA) was used as the human skin fibroblast cell line. The Dulbecco’s Modified Eagle Medium (DMEM) (Sigma Aldrich) was used as the cell culture medium. The 3-(4,5-dimethylthiazol-2-yl)-2,5-diphenyl tetrazolium bromide (MTT) (Sigma Aldrich) and dimethylsufoxide (DMSO, Darmstadt, Germany) were used as the MTT reagent and formazan solvent.

### 2.2. Synthesis of PEDOT:PSS

PEDOT:PSS was synthesized as previously reported by Sakunpongpitiporn et al., 2019 [28]. A total of 5.0 g (4.95 mL) of PSS was added to DI water (100 mL) under continuous stirring for 1 h. A total of 0.5 g (0.38 mL) of EDOT was added to the above solution at the EDOT:PSS weight ratio of 1:11 under continuous stirring for 1 h. Next, 1.67 g of SP was added into the solution at the EDOT:SP mole ratio of 1:2 under stirring at room temperature for 24 h. Finally, the PEDOT:PSS precipitate was rinsed with the mixed solvent of C_3_H_6_O (30 mL) and CH_3_OH (200 mL) and centrifuged at 6000 rpm. The PEDOT:PSS precipitate was dried in an oven at 70 °C for 8 h.

### 2.3. Preparation of Sonidegib/PEDOT:PSS

A total of 0.01 g of PEDOT:PSS was dissolved in 2 mL of DI water under continuous stirring at room temperature for 7 days. Then, 0.2 g Sonidegib was dissolved in 5 mL of ethanol and stirred at room temperature for 30 min. The PEDOT:PSS solution was added to the Sonidegib solution under stirring at room temperature for 30 min to obtain the Sonidegib/PEDOT:PSS solution.

### 2.4. Fabrication of Sonidegib/PEDOT:PSS/Gelatin Hydrogel Matrices

The BG and PG powders of the concentrations of 5% *v*/*v* (1.30 g), 6% *v*/*v* (1.56 g), and 7% *v*/*v* (1.82 g) were each dissolved in 13 mL DI water under continuous stirring at 40 °C for 30 min. Next, the gelatin solutions were cooled down from 40 to 27 °C, and the Sonidegib/PEDOT:PSS solution was added to the gelatin solutions under stirring for 10 min. Finally, the Sonidegib/PEDOT:PSS/gelatin solutions were cast into Petri dishes.

### 2.5. Characterizations

To determine the degrees of swelling and weight losses, the gelatin and PEDOT:PSS/gelatin hydrogels (2 mm in diameter and 3 ± 0.2 mm in thickness) were immersed in the PBS buffers at 37 ± 0.5 °C (pH of 7.4, human blood or pH of 5.5, human skin) with and without the electric voltages [29]. An electric field of various voltages (3, 6 V) was applied to the hydrogel matrices by a cathode electrode connected to a power supply (Tektronix, Beaverton, OR, USA, PS 280) and attached on top of the sample for 5 h (swelling equilibrium time). Each test was carried out using 3 samples. Each sample was weighed at various times for up to 5 h (swelling equilibrium time). The degree of swelling and weight losses of the gelatin and PEDOT:PSS/gelatin hydrogels were calculated by Equations (1) and (2) [30]:

(1) $$Degree\;of\;swelling\;(\%)=\frac{M_{s}\;-\;M_{d}}{M_{d}}\;\times \;100$$

(2) $$Weight\;Losses\;(\%)=\frac{M_{i}\;-\;M_{d}}{M_{i}}\;\times \;100$$

where M_s_ is the weight of a swollen sample (g), M_d_ is the weight of the swollen sample after drying at 27 °C (g), and M_i_ is the initial weight of the sample (g).

To determine the mesh sizes (ξ) and crosslinking densities (ρ_x_) of the gelatin and PEDOT:PSS/gelatin hydrogels, each sample was weighed in air and heptane, and then the sample was immersed in the PBS buffers (pH of 7.4, human blood or pH of 5.5, human skin) with and without electric voltages. An electric field of various voltages (3, 6 V) was applied to the hydrogel matrices by a cathode electrode connected to a power supply (Tektronix, PS 280) and attached on top of the sample for 5 h. Each test was carried out using 3 samples. After that, each sample was dried at 27 °C for 12 h and weighed in air and heptane again. The mesh sizes (ξ) and crosslinking densities (ρ_x_) were calculated by Equation (3) [31], Equation (4), and Equation (5) [32]:

(3) $$\frac{1}{{\overset{\text{—}}{M}}_{c}}\;=\;\frac{2}{{\overset{\text{—}}{M}}_{n}}\;-\;\frac{\frac{\overset{\text{—}}{\nu }}{V_{1}}\left[\ln\left(1\;-\;\upsilon_{2,s}\right)\;+\;\upsilon_{2,s}\;+\;\chi \upsilon_{2,s}^{2}\right]}{\upsilon_{2,r}\left[{\left(\frac{\upsilon_{2,s}}{\upsilon_{2,r}}\right)}^{1/3}\;-\;\frac{1}{2}\left(\frac{\upsilon_{2,s}}{\upsilon_{2,r}}\right)\right]}$$

(4) $$\xi \;=\upsilon_{2,s}^{-1/3}{\left[C_{n}\left(\frac{{2\overset{\text{—}}{M}}_{c}}{{\overset{\text{—}}{M}}_{r}}\right)\right]}^{1/2}l$$

(5) $$\rho_{x}\;=\frac{1}{\bar{\nu }{\overset{\text{—}}{M}}_{c}}$$

where υ_2,s_ is the swollen equilibrium polymer volume fraction, υ_2,r_ is the relaxed equilibrium polymer volume fraction, $\bar{\upsilon }$ is the specific volume of gelatin (0.741 cm^3^/g [33]), χ is the Flory Huggins interaction parameter of gelatin and water (0.497 [33]), V_1_ is the molar volume of DI water (18 cm^3^/mole), ${\overset{\text{—}}{M}}_{n}$ is the number-average molecular weight of the primary gelatin chains (50,000 and 41,000 g/mole for BG and PG, respectively), ${\overset{\text{—}}{M}}_{c}$ is the molecular weight between crosslinking (g/mol), C_n_ is Flory’s characteristic ratio of gelatin (8.26 [34]), *l* is the carbon-carbon bond length (1.543 Å), and ${\overset{\text{—}}{M}}_{r}$ is the molecular weight of the gelatin repeating units (114.8 g/mol [34]).

A UV-visible spectrometer (TECAN, Männedorf, Switzerland, Infinite M200) was used to determine the characteristic peak and the amounts of Sonidegib release and release-permeation at various times. The instrument was operated between 230 and 1000 nm, where the Sonidegib absorbance peak was identified to be located at 276 nm [35]. A calibration curve was obtained between the measured absorbance intensities and Sonidegib concentrations.

To determine the actual drug contents, the Sonidegib/BG and Sonidegib/PEDOT:PSS/BG hydrogels were dissolved in the 15 mL PBS buffer for 7 days. A total of 0.1 mL of the solution was withdrawn and diluted with 3 mL of the PBS buffer. The concentrations of Sonidegib in the solutions were measured and determined by the UV-visible spectrometer at a wavelength of 276 nm, using the calibration curve.

In vitro Sonidegib drug release and in vitro release-permeation experiments were investigated by the modified Franz diffusion cell [36]. The modified Franz diffusion cell consisted of 2 parts: the donor and acceptor parts. The receptor part contained a PBS buffer solution (pH of 7.4, human blood or pH of 5.5, human skin) at 37 °C. The Sonidegib/BG and Sonidegib/PEDOT:PSS/BG hydrogels were placed over a nylon net or a pig belly skin for in vitro drug release and in vitro drug release-permeation in the donor part. An electric field of various voltages (3, 6 V) was applied to the hydrogel matrices by a cathode electrode connected to a power supply and attached on top of the sample for 24 h, where the resultant currents were 0.12–3.14 μA. Sonidegib was released or release-permeated from the hydrogels into the PBS buffer. A total of 0.1 mL of solution was collected from the receptor part and replaced with the same volume of fresh PBS at various times. The amounts of Sonidegib release were measured by the UV-visible spectrometer at a wavelength of 276 nm.

The cytotoxicity of the 5% *v*/*v* BG and PEDOT:PSS/5% *v*/*v* BG hydrogels was investigated by the MTT assay (ISO 10993-5). Each test was carried out using 3 samples. A cell suspension of 1 × 10^5^ cells/mL CRL-2522 (human skin fibroblast cells) in a DMEM complete medium was seeded into the 96-well plate. It was incubated at 37 ± 1 °C, 5 ± 0.1% CO_2,_ and at 95 ± 5 relative humidity for 24 ± 2 h to obtain confluent monolayers of cells [29]. The DMEM completed medium was replaced with the extracts of: the blank (the media without a test specimen); the negative control (‘Thermanox’, Nunc (Thermo Fisher Scientific, Roskilde, Denmark) coverslip was used as a negative control material, where the surface-area-to-volume extraction ratio was 6 cm^2^/mL); the positive control (‘Polyurethane film containing 0.1% Zinc diethyldithiocarbamate (ZDEC) RM-A’ was used as a positive control material, where the surface-area-to-volume extraction ratio was 3 cm^2^/mL); and the tested specimens (the mass-to-volume extraction ratio of 0.1 g/ml was used).

All of the tested specimens were extracted at 37 ± 1 °C for 24 ± 2 h. The extracts of the tested specimens were filtered with a 0.22 μm syringe filter prior to use. The cells were incubated further for 24 ± 2 h.

After incubation, the viable cells were stained with MTT and incubated further for 2 h. Then MTT was removed and DMSO was added to each well. The absorbance was measured using a microplate reader at 570 nm.

To study the release mechanism characteristics of Sonidegib from gelatin and PEDOT:PSS/gelatin hydrogels, the power law model was used to investigate the Sonidegib release mechanism. The cumulative Sonidegib release amounts (*y* axis) vs. times (*x* axis) were identified by the Korsmeyer and Peppas model [37] as in Equation (6):

(6) $$\frac{M_{t}}{M_{\infty }}\;=\;{\mathrm{kt}}^{n}$$

where M_t_/M_∞_ is the fractional Sonidegib release amount, t is the release time, k is a kinetic constant characteristic, and n is the apparent diffusional scaling exponent indicating the release mechanism, respectively. When n is equal to 0.5, the equation above becomes the Higuchi equation [38]. The diffusion coefficient (D) can be calculated from the Higuchi relation as in Equation (7) [38]:

(7) $$Q=\;\frac{M_{t}}{A}\;=\;{2C}_{0}{\left(\frac{\mathrm{Dt}}{\pi }\right)}^{1/2}$$

where M_t_ is the amount of drug released (g), A is the diffusion coefficient area (cm^2^), C_0_ is the initial drug concentration in the hydrogels (g/cm^3^), and D is the diffusion coefficient of the drug (cm^2^/s).

A Fourier transform infrared spectrometer (FT-IR, Nicolet, iS5, Bangkok, Thailand), a simultaneous thermal analyzer (STA (Perkin Elmer, Pyris Diamond, Waltham, MA, USA), an X-ray photoelectron spectrometer (XPS (Kratos, Axis Ultra DLD, Manchester, UK)), and a UV-visible spectrometer (UV-vis, Infinite M200, TECAN, Männedorf, Switzerland)) were used to characterize PEDOT:PSS, Sonidegib, Sonidegib/PEDOT:PSS, gelatin hydrogels, Sonidegib/gelatin hydrogels, and Sonidegib/PEDOT:PSS/gelatin hydrogels, as described in the Supplementary Materials. The statistical analysis and data are given in the Supplementary Materials.

## 3. Results and Discussion

### 3.1. Characterization

**Figure 1:**
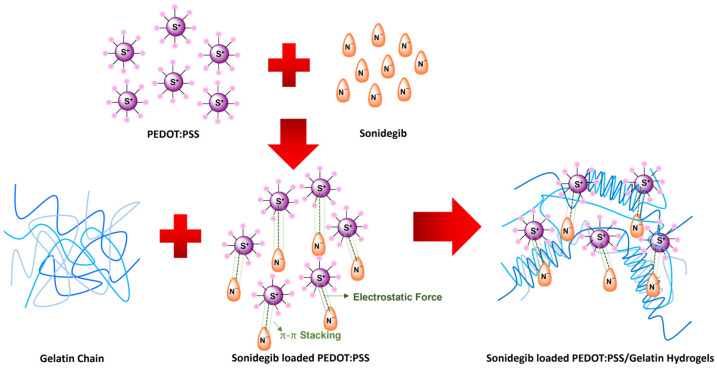
The proposed structure of Sonidegib/PEDOT:PSS and transformation of gelatin from random coils to triple helices.

PEDOT:PSS was synthesized via chemical oxidative polymerization [28]. The PEDOT positively charged chains interacted with the dopant PSS negative charges via the electrostatic force. Sonidegib was an anionic drug [39] interacting with the PEDOT chain via the electrostatic force and the π-π stacking between aromatic rings of PEDOT:PSS to form the Sonidegib/PEDOT:PSS, as shown in Figure 1. At a high temperature (40 °C), the gelatin was in a sol state with a random coil conformation. At a lower temperature, 30 °C, as the Sonidegib/PEDOT:PSS was added to the gelatin solution, the gelatin chains transformed from the sol state with a random coil conformation to triple helices, as shown in Figure 1 [21].

#### 3.1.1. FTIR and TGA

**Figure 2:**
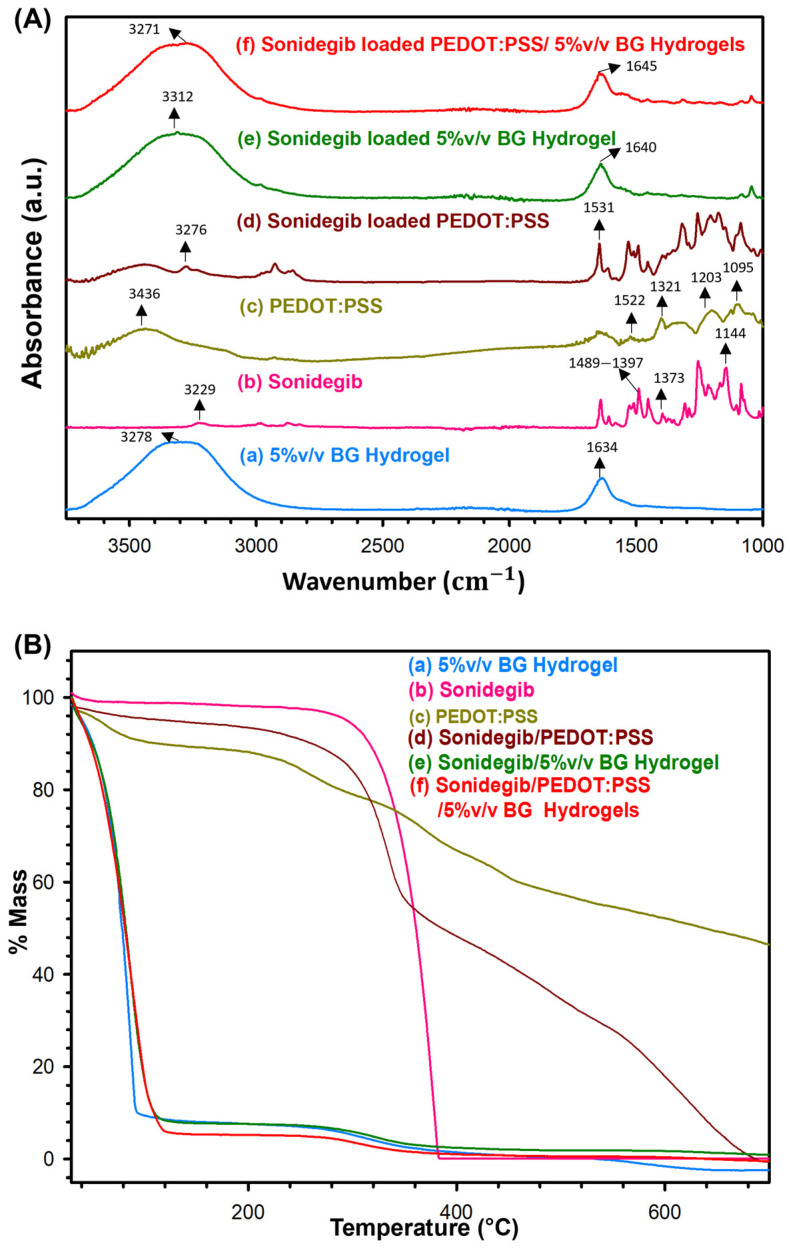
Characterizations: (**A**) FTIR Spectra; (**B**) TGA Thermograms.

The FTIR spectra of the 5% *v*/*v* BG hydrogel, Sonidegib, PEDOT:PSS, Sonidegib/PEDOT:PSS, Sonidegib/5% *v*/*v* BG hydrogel, and Sonidegib/PEDOT:PSS/5% *v*/*v* BG hydrogels are illustrated in Figure 2A and Table S1. In Figure 2(Aa), the 5% *v*/*v* BG hydrogel spectrum indicates the peaks of 3278 and 1634 cm^−1^ due to overlapping between the O-H and N-H stretching vibrations, and C=O stretching of amide I (Gly-Pro-Hyp tripeptide chains), respectively [40]. Additionally, the peak at 1634 cm^−1^ can be attributed to the triple helices in BG hydrogel as generated in the cooling process [41]. The Sonidegib FTIR spectrum, as shown in Figure 2(Ab), illustrates the peaks at 3229,1489–1397, 1373, and 1144 cm^−1,^ which can be attributed to the N-H stretching, CH_3_ stretching, aromatic C-C, and C-H stretching, respectively [39]. The PEDOT:PSS FTIR spectrum (Figure 2(Ac)) demonstrates the peaks at 3436, 1522, and 1321 cm^−1,^ indicating the O-H, C=C, C-C stretching vibrations, whereas the peaks at 1203 and 1095 cm^−1^ imply the S=O symmetric stretching and asymmetric stretching vibrations, respectively [28]. The Sonidegib/PEDOT:PSS spectrum, as shown in Figure 2(Ad), shows the peak shifts from 3229 (pristine Sonidegib) to 3276 cm^−1^, and 1522 (PEDOT:PSS) to 1531 cm^−1^; they can be assigned to the N-H stretching and C=C stretching, respectively. These peak shifts suggest that Sonidegib was successfully loaded into and reacted with PEDOT:PSS. The spectrum of Sonidegib/5% *v*/*v* BG hydrogel in Figure 2(Ae) demonstrates the peak shifts from 3278 to 3312 cm^−1^ and 1634 to 1640 cm^−1^ when compared to the 5% *v*/*v* BG hydrogel. These peak shifts suggest that Sonidegib was incorporated into the 5% *v*/*v* BG hydrogel, in which the 5% *v*/*v* BG hydrogel conformation consisted of the triple helix. The FTIR spectrum of Sonidegib/PEDOT:PSS/5% *v*/*v* BG hydrogel in Figure 2(Af) shows the peak shifts from 3278 to 3271 cm^−1^ and 1634 to 1645 cm^−1^ when compared to the 5% *v*/*v* BG hydrogel. These peak shifts suggest that Sonidegib was successfully loaded into and interacted with PEDOT:PSS and the 5% *v*/*v* BG hydrogel, whereas the 5% *v*/*v* BG hydrogel still retained the triple helix conformation.

The TGA thermograms of 5% *v*/*v* BG hydrogel, Sonidegib, PEDOT:PSS, Sonidegib/PEDOT:PSS, Sonidegib/5% *v*/*v* BG hydrogel, and Sonidegib/PEDOT:PSS/5% *v*/*v* BG hydrogels are shown in Figure 2B, and the data are shown in Tables S2 and S3. The 5% *v*/*v* BG hydrogel possesses two decomposition stages: 40–140 °C can be attributed to the water evaporation; 140–700 °C is due to the cleavage of bonds of protein chains associated with the helical structure [42]. The T_d,onset_ of the 5% *v*/*v* BG hydrogel is 277 °C [43]. Sonidegib displays only one decomposition stage at 180–500 °C, whereas T_d,onset_ is at 347 °C. PEDOT:PSS reveals four decomposition stages: 40–140 °C assigned to the water evaporation; 140–300 °C, the decomposition of the PEDOT side chain; 300–400 °C, the decomposition of PSS; and 400–700 °C, the decomposition of PEDOT [28]. T_d,onset_ (%mass losses) of PEDOT side chain, PSS, and PEDOT are at 221 °C (11.23%), 315 °C (12.71%), and 456 °C (29.74%), respectively. The Sonidegib/PEDOT:PSS possesses four decomposition stages: 40–180 °C, the evaporation of water; 180–400 °C, the overlapping decompositions of the PEDOT side chain and Sonidegib; 400–540 °C, the decomposition of PSS; and 540–700 °C, the decomposition of PEDOT. The T_d,onset_ of the overlapping PEDOT side chain, and Sonidegib, PSS, and PEDOT are 300 °C, 427 °C, and 571 °C, whereas the %mass losses are 46.54%, 17.69%, and 29.33%, respectively. The T_d,onset_ value of Sonidegib/PEDOT:PSS when compared to PEDOT:PSS shifted to a higher temperature from 456 to 571 °C, whereas the % mass loss of the overlapping PEDOT side chain and Sonidegib significantly increased from 11.23% to 46.54%. These results suggest that Sonidegib was successfully loaded and interacted with PEDOT. The Sonidegib/5% *v*/*v* BG hydrogel possesses two decomposition stages similar to the 5% *v*/*v* BG hydrogel. The T_d,onset_ value of Sonidegib/5% *v*/*v* BG hydrogel shifted from 277 to 260 °C, resulting from Sonidegib being incorporated into the 5% *v*/*v* BG hydrogel. The Sonidegib/PEDOT:PSS/5% *v*/*v* BG hydrogel possesses two decomposition stages similar to the 5% *v*/*v* BG hydrogel. The T_d,onset_ value of the Sonidegib/PEDOT:PSS/5% *v*/*v* BG hydrogel shifted from 277 to 266 °C. The possible reason is that Sonidegib/PEDOT:PSS was successfully loaded and incorporated into the 5% *v*/*v* BG hydrogels, consistent with the FTIR results.

#### 3.1.2. XPS

**Figure 3:**
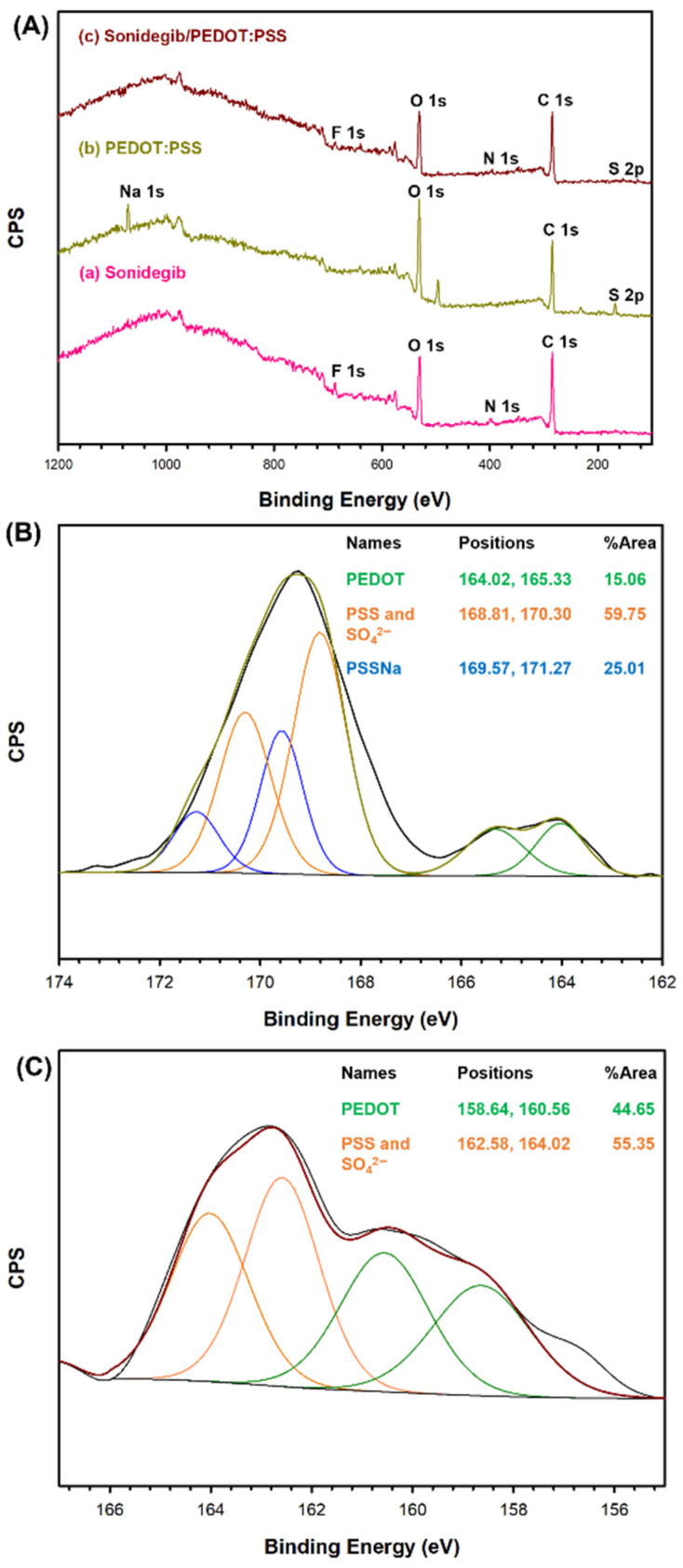
XPS spectra: (**A**) Survey scan high resolution XPS spectra of S 2p of: (**B**) PEDOT:PSS; and (**C**) Sonidegib/PEDOT:PSS. The green, orange, and blue lines are the percentages of PEDOT, PSS and SO_4_^2−^, and PSSNa, respectively.

The survey scan XPS spectra and element compositions of Sonidegib, PEDOT:PSS, and Sonidegib/PEDOT:PSS are in Figure 3 and Table S4, respectively. The Sonidegib spectrum, as shown in Figure 3(Aa), displays O 1s (27.08%), C 1s (67.89%), N 1s (1.89%), and F 1s (3.14%). The PEDOT:PSS spectrum, as shown in Figure 3(Ab), exhibits O 1s (34.25%), C 1s (59.18%), S 2p (2.72%), and Na 1s (3.85%) [28]. The Sonidegib/PEDOT:PSS spectrum, as shown in Figure 3(Ac), is similar to the Sonidegib and PEDOT:PSS spectra. However, the Sonidegib/PEDOT:PSS spectrum shows distinctively different peaks of N 1s (1.32%) and F 1s (0.38%), indicating the Sonidegib and PEDOT:PSS characteristics.

The high-resolution XPS spectra of PEDOT:PSS and Sonidegib/PEDOT:PSS are shown in Figure 3B and Figure 3C, respectively. The S 2p of PEDOT:PSS, as shown in Figure 3B, can be identified at 164.02 eV, 165.33 eV for PEDOT, 168.81 eV, 170.30 eV for PSS and SO_4_^2−^, and 169.57 eV, 171.27 eV for PSSNa. The %S are 15.06% for PEDOT, 59.75% for PSS and SO_4_^2−^, and 25.01% for PSSNa [28]. The S 2p of Sonidegib/PEDOT:PSS, as shown in Figure 3C, is located at 158.64 eV, 160.56 eV for PEDOT, and 162.58, 164.02 eV for PSS and SO_4_^2−^. The %S are 44.65% for PEDOT, and 55.35% for PSS and SO_4_^2−^. The Sonidegib/PEDOT:PSS spectrum indicates the shifts to lower binding energy when compared to that of PEDOT:PSS. These results suggest that Sonidegib was successfully loaded into and interacted with PEDOT:PSS.

In the case of the Sonidegib/PEDOT:PSS/5% *v*/*v* BG hydrogel, all of the FTIR, TGA, and XPS results suggest that Sonidegib was successfully loaded into and interacted with PEDOT:PSS via the electrostatic force, π-π stacking, and hydrogen bonding. The 5% *v*/*v* BG hydrogel thermogram indicates via temperatures that the physical crosslinking changed the BG conformation from the arbitrary coil to the triple helix, as shown in Figure 1.

#### 3.1.3. Swelling and Crosslinking Density

The % swelling, weight losses, mesh sizes, and crosslinking densities of gelatin hydrogels are tabulated, as shown in Table 1. For the effect of gelatin types (BG and PG), the % swelling values of the 5% *v*/*v* BG and PG hydrogels at the pH of 7.4 are 336.40 ± 29.86% and 111.39 ± 1.85% (*p* < 0.05), whereas the mesh sizes are 16.4 ± 3.7 Å and 9.7 ± 1.5 Å (*p* < 0.05), respectively. Because the BG hydrogel (isoelectric point, pI = 5) had a higher amount of negative charges, this resulted in a higher electrorepulsive force than the PG hydrogel (pI = 9) [44]. At higher gelatin concentrations (BG and PG), the % swelling, weight loss, and mesh size decreased due to the higher entanglement, resulting in lower free volumes to adsorb water within the smaller mesh sizes [45]. At lower pH values, the % swelling, weight loss, and mesh size decreased because of the lower, negatively-charged BG chains (pI = 5), resulting in a smaller electrorepulsive force [46]. At higher electrical voltages, the % swelling, weight loss, and mesh size increased due to the higher electrorepulsive force between the cathode electrode and the BG chains, inducing the higher mesh sizes and the matrix expansion [29].

For the effect of PEDOT:PSS, the % swelling and mesh size increased from 336.40 ± 29.86% to 384.73 ± 2.18% (*p* < 0.05) and 16.4 ± 3.7 Å to 19.9 ± 4.0 Å (*p* < 0.05), respectively. As PEDOT:PSS is a hydrophilic polymer, it can be easily incorporated into the BG chains, resulting in higher mesh sizes along with the matrix expansion [47]. For the effect of electric voltages on PEDOT:PSS/5% *v*/*v* BG, the % swelling and mesh size significantly increased due to various electrostatic interactions: the negatively charged PSS molecules and the BG chains; the cathode electrode and the PSS molecules; and the cathode electrode and the BG chains [48].

#### 3.1.4. Morphology

**Figure 4:**
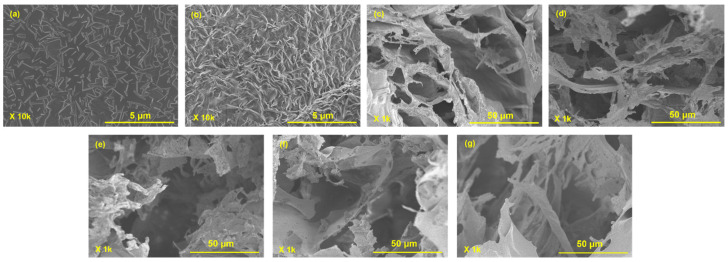
SEM images of: (**a**) PEDOT:PSS; (**b**) Sonidegib/PEDOT:PSS; (**c**) 5% *v*/*v* PG; (**d**) 5% *v*/*v* BG; (**e**) 5% *v*/*v* BG at E = 6 V; (**f**) PEDOT:PSS/5% *v*/*v* BG; and (**g**) PEDOT:PSS/5% *v*/*v* BG at E = 6 V.

The surface SEM images of PEDOT:PSS and Sonidegib/PEDOT:PSS are shown in Figure 4a,b. The PEDOT:PSS image shows a smooth surface with irregular conformations [49], whereas the Sonidegib/PEDOT:PSS surface shows a higher roughness with fibrous shapes. These results imply that Sonidegib was loaded and incorporated into PEDOT:PSS, consistent with the FTIR, TGA, and XPS results. The cross-section SEM images are shown in Figure 4c–g. Here, the images show interconnected pores and porous structures [50]. For the effect of gelatin types (Figure 4c,d), the 5% *v*/*v* BG hydrogel displays larger pore sizes than the 5% *v*/*v* PG hydrogel because the BG hydrogel (pI = 5) has higher negative charges, resulting in a higher electrorepulsive force than the PG hydrogel (pI = 9) [44]. Under electrical voltages (Figure 4d,e), the pore size increased due to the electrorepulsive force between the cathode electrode and the BG chains. For the effect of PEDOT:PSS (Figure 4d,f), the pore size increased because PEDOT:PSS was incorporated with the BG chains, leading to partial matrix expansion [47]. Under the effect of electric voltages on PEDOT:PSS/5% *v*/*v* BG (Figure 4f,g), the pore size increased due to the higher electrorepulsive force. The possible reasons are the electrostatic interactions between the negatively charged PSS molecules and the BG chains, the cathode electrode and the PSS molecules, and the cathode electrode and the BG chains [48]. All image results support the swelling behaviors as tabulated in Table 1.

#### 3.1.5. Cytotoxicity

The cytotoxicity of 5% *v*/*v* BG and PEDOT:PSS/5% *v*/*v* BG hydrogels was examined by the MTT assay. The %cell viability of the 5% *v*/*v* BG and PEDOT:PSS/5% *v*/*v* BG hydrogels are 88 ± 1% (*p* < 0.05) and 87 ± 1% (*p* < 0.05), respectively. The %cell viability is higher than 70%, suggesting that all hydrogels were non-toxic to the human skin [51].

### 3.2. Release Behaviors

#### 3.2.1. Actual Sonidegib Loaded Amounts

The actual Sonidegib loaded amounts into the gelatin and PEDOT:PSS/gelatin hydrogels were investigated by UV-vis, as shown in Table 2 and Table 3. The actual Sonidegib loaded amounts and percentages of Sonidegib loading efficiency for 5% *v*/*v* PG, 5% *v*/*v* BG, 6% *v*/*v* BG, and 7% *v*/*v* BG hydrogels were 19.5 ± 0.2 and 97.5%, 19.7 ± 0.1 and 98.5%, 19.3 ± 0.2 and 96.5%, and 19.3 ± 0.3 mg and 96.5%, (*p* < 0.05), respectively. The actual Sonidegib loaded amounts into the PEDOT:PSS/5% *v*/*v* BG hydrogels and percentages of Sonidegib loading efficiency were 19.6 ± 0.2 mg and 98.0% (*p* < 0.05), respectively.

#### 3.2.2. Effect of Gelatin Types

**Figure 5:**
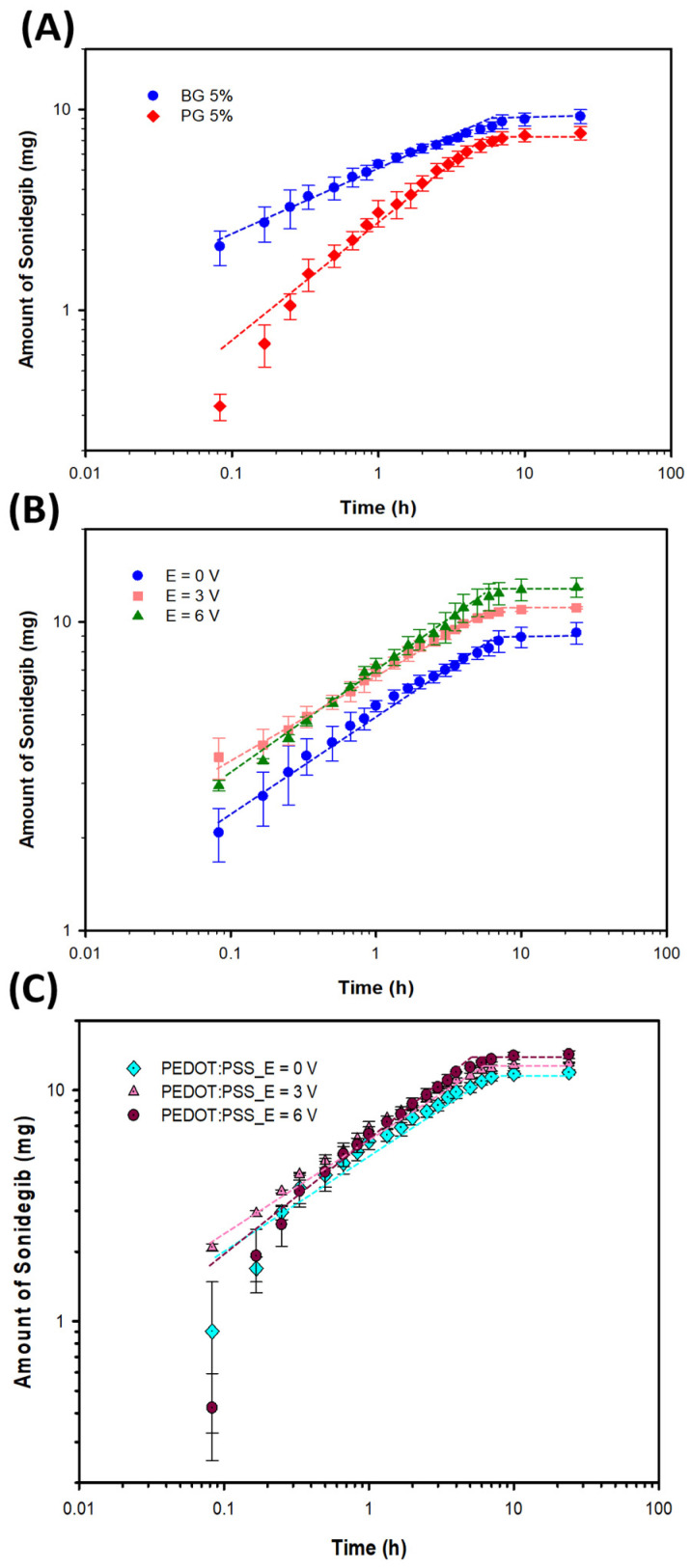
Amounts of Sonidegib released from the BG and PG gelatin hydrogels under the effects of: (**A**) gelatin types (BG and PG); (**B**) electric fields (5% *v*/*v* BG); and (**C**) PEDOT:PSS/5% *v*/*v* BG. The dashed lines indicate the trend lines.

The amounts of Sonidegib released from the 5% *v*/*v* gelatin hydrogels and the release data of the PG and BG gelatins are shown in Figure 5A, Figure S2A, and Table 2, respectively. The n values of 5% *v*/*v* PG and 5% *v*/*v* BG are 0.34 and 0.31, respectively. This indicates that the Sonidegib release was primarily from the concentration gradient (Fickian diffusion) [52]. For the 5% *v*/*v* PG and 5% *v*/*v* BG, the % Sonidegib releases were 38.10% and 46.10% (*p* < 0.05), whereas the corresponding times to equilibrium were 6.0 h and 3.5 h, respectively. Sonidegib was an anionic drug, whereas the BG hydrogel (pI = 5) possessed higher negative charges, resulting in the higher electrorepulsive force between the drug and the matrix [44]. For 5% *v*/*v* PG, the amount of Sonidegib released decreased due to the electroattractive force between the negatively charged drug and the positively charged PG matrix.

#### 3.2.3. Effect of Gelatin Concentrations

The amounts of Sonidegib released from the BG hydrogels and the release data of various BG concentrations are shown in Figure S1a,b and Table 2, respectively. The n values of 5% *v*/*v* BG, 6% *v*/*v* BG, and 7% *v*/*v* BG are 0.31, 0.46, and 0.54. For 5% *v*/*v* BG and 6% *v*/*v* BG, the n values are less than 0.5, which can be classified as the Fickian diffusion as controlled by the concentration gradient [52]. At 7% *v*/*v* BG, with n > 0.5, this can be classified as the anomalous transport or non-Fickian diffusion as controlled by the concentration gradient and swelling [52]. At higher BG concentrations, the % Sonidegib release and diffusion coefficient decreased from 46.10% to 30.90% (*p* < 0.01) and from 4.80 × 10^−13^ to 1.76 × 10^−13^ cm^2^s^−1^ whereas the time to equilibrium increased from 3.5 to 7.0 h, respectively. At higher concentrations, there were more entanglements present, resulting in lower mesh sizes and longer times to reach equilibrium [53].

#### 3.2.4. Effect of Electrical Voltages

The amounts of Sonidegib released from the 5% *v*/*v* BG hydrogel and the release data under electrical voltages are shown in Figure 5B, Figure S2B, and Table 2, respectively. The n values of 5% *v*/*v* BG at 0, 3, and 6 V are 0.31, 0.27, and 0.34. The n values here are lower than 0.5, which indicate the Fickian diffusion or concentration gradient [52]. At higher electrical voltages, the % Sonidegib release and diffusion coefficient increased from 46.10% to 64.67% (*p* < 0.01) and from 4.80 × 10^−13^ to 9.60 × 10^−13^ cm^2^s^−1^ in which the time to equilibrium decreased from 3.5 to 2.5 h, respectively. At higher electrical voltages, the amount of Sonidegib release increased due to the higher electroosmosis and electrorepulsive forces between the negatively charged drug and the cathode electrode, and the negatively charged drug and the BG matrix expansion.

#### 3.2.5. Effect of PEDOT:PSS

The amounts of Sonidegib released from the PEDOT:PSS/5% *v*/*v* BG hydrogel and release data at various electrical voltages are exhibited in Figure 5C, Figure S2C, and Table 2, respectively. The n values of the 5% *v*/*v* BG and PEDOT:PSS/5% *v*/*v* BG hydrogels at 0 V are 0.31 and 0.52. Adding PEDOT:PSS improved the swelling behavior, resulting in the n value being greater than 0.5 [54]. The n values of the PEDOT:PSS/5% *v*/*v* BG hydrogel at 0, 3, and 6 V are 0.52, 0.42, and 0.65, respectively. At zero and 6 V, the n values are higher than 0.5, which can be classified as the anomalous transport or non-Fickian diffusion as controlled by the concentration gradient and partial swelling [52].

Adding PEDOT:PSS, the amount of Sonidegib release increased from 46.10% to 59.68% (*p* < 0.01), whereas the time to equilibrium increased from 3.5 to 6.0 h. The amount of Sonidegib release increased when adding PEDOT:PSS because PEDOT:PSS is a hydrophilic polymer, inducing the matrix mesh size to increase [54]. At higher electrical voltages, the % Sonidegib release and diffusion coefficient increased from 59.68% to 71.29% (*p* < 0.01) and from 7.56 × 10^−13^ to 1.05 × 10^−12^ cm^2^s^−1^, respectively. Under the electrical voltages, the amount of Sonidegib release increased due to the higher electroosmosis and electrorepulsive forces. The electrorepulsive force resulted from the cathode electrode and the PSS molecules, the cathode electrode and the BG chains leading to the matrix expansion, and the negatively charged PSS molecules and the BG chains [48].

### 3.3. Release-Permeation Behaviors

#### 3.3.1. Effect of pH Values

**Figure 6:**
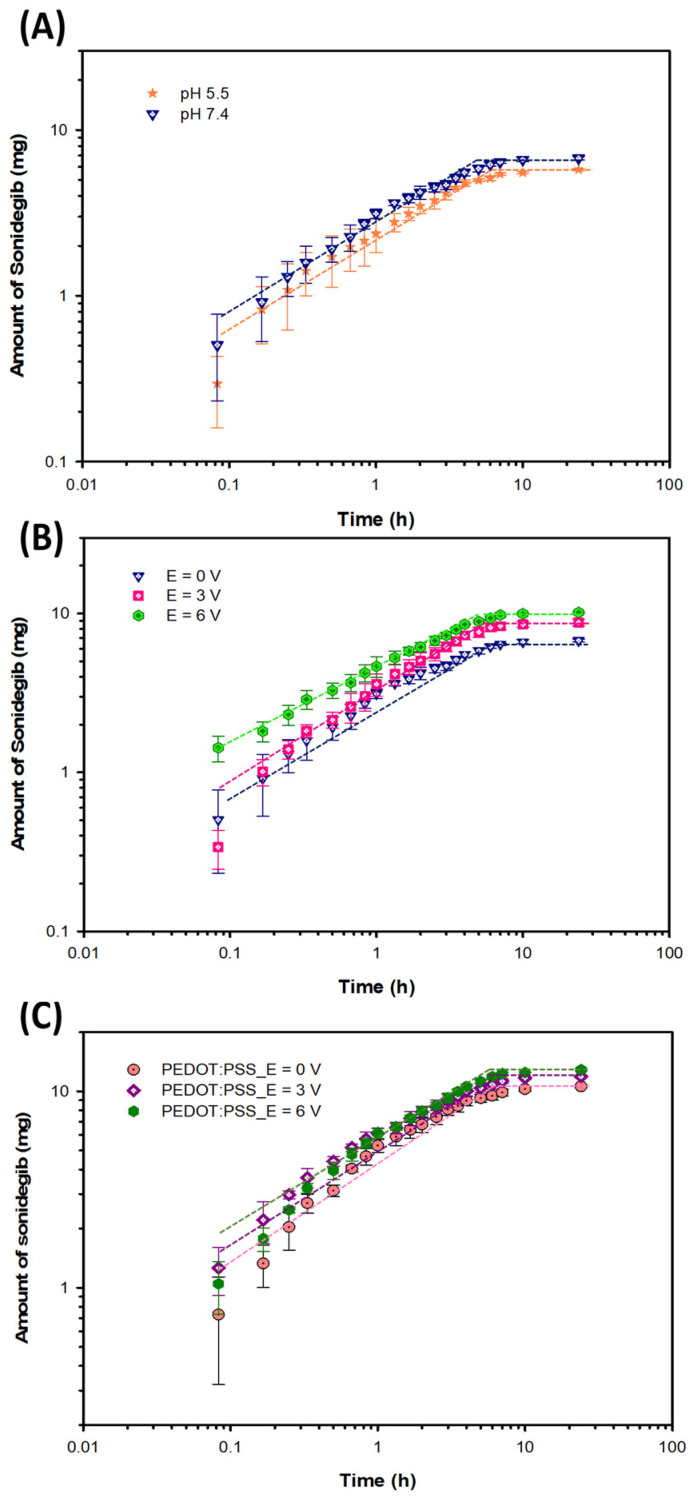
Amounts of Sonidegib released-permeated from the 5% *v*/*v* BG hydrogel through the pig skin under the effects of: (**A**) pH values; (**B**) electric fields; and (**C**) PEDOT:PSS. The dashed lines indicate the trend lines.

The amounts of Sonidegib released-permeated from 5% BG and the release-permeation data at various pH values are shown in Figure 6A, Figure S3A, and Table 3, respectively. The n values of 5% BG at the pH values of 5.5 and 7.4 are 0.58 and 0.55, respectively. The n values are higher than 0.5, indicating the anomalous transport or non-Fickian diffusion as controlled by the concentration gradient and swelling [52]. The % Sonidegib release-permeation of 5%BG at the pH values of 5.5 and 7.4 are 28.83%, 33.81% (*p* < 0.01), respectively, whereas the times to equilibrium are 6.0 h and 5.0 h. At lower pH values, the amount of sonidegib release-permeation decreased because BG (pI = 5) possessed the lower negative charges, resulting in the smaller electrorepulsive force between the drug and the matrix.

#### 3.3.2. Effect of Electrical Voltages

The amounts of Sonidegib released-permeated from 5% BG and the released-permeation data under various electrical voltages are shown in Figure 6B, Figure S3B, and Table 3, respectively. The n values of 5% BG at E = 0, 3, and 6 V are 0.55, 0.64, and 0.46, respectively. The Sonidegib release-permeation was mainly controlled by the concentration gradient and partial swelling [52]. At higher electrical voltages, the % Sonidegib release-permeation and diffusion coefficient increased from 33.81% to 50.78% (*p* < 0.05) and from 2.34 × 10^−13^ to 5.42 × 10^−13^ cm^2^s^−1^ whereas the time to equilibrium decreased from 5.0 to 4.0 h, respectively. At higher electrical voltages, the amount of Sonidegib release-permeation increased due to the higher electroosmosis and the electrorepulsive forces between the negatively charged drug and the cathode electrode, and the negatively charged drug and the matrix. These forces acted as the driving forces for the drug to permeate through the porcine skin.

#### 3.3.3. Effect of PEDOT:PSS

The amounts of Sonidegib released-permeated from PEDOT:PSS/5% BG and the release-permeation data at various electrical voltages are shown in Figure 6C, Figure S3C, and Table 3, respectively. The n values of PEDOT:PSS/5% BG at E = 0, 3, 6 V are 0.56, 0.53, 0.56, respectively. These values are greater than 0.5, indicating the anomalous transport or non-Fickian diffusion as controlled by the concentration gradient and swelling [52]. Upon adding PEDOT:PSS, the % Sonidegib release-permeation increased from 33.81% to 52.94% (*p* < 0.05), because PEDOT:PSS can be easily incorporated into the BG matrix, resulting in a larger mesh size [54]. Under applied electrical voltages, the % Sonidegib release-permeation and diffusion coefficient increased from 52.94% to 64.38% (*p* < 0.001) and 4.31 × 10^−13^ to 8.59 × 10^−13^ cm^2^s^−1,^ while the time to equilibrium decreased from 6.0 to 4.5 h, respectively. At higher electrical voltages, the amount of Sonidegib release-permeation increased because of the higher electroosmosis and electrorepulsive forces. The electrorepulsive forces occurred from the cathode electrode and the PSS molecules, the cathode electrode and the BG chains, and the negatively charged PSS molecules and the BG chains.

The overall results suggest that the BG and PEDOT:PSS/BG hydrogels are applicable to be used as the matrices in which the rate, amount, and duration of the transdermal Sonidegib delivery can be controlled via iontophoresis.

In the in vitro release permeation study, the amounts of Sonidegib permeated from bovine skin (BG) hydrogel at E = 0, 3, and 6 V (pH = 7.4) were 6.76, 8.78, and 10.16 mg, respectively. This indicated that the amount of Sonidegib release-permeation increased, resulting from the higher electroosmosis and the electrorepulsive forces between the negatively charged drug and the cathode electrode, and the negatively charged drug and the BG matrix. With the addition of PEDOT:PSS, the amounts of Sonidegib permeated from PEDOT:PSS/BG hydrogels increased from 10.16 mg to 10.58 mg (E = 0 V), 11.89 mg (E = 3 V), and 12.88 mg (E = 6 V). This suggested that the PEDOT:PSS incorporated into the BG hydrogel resulted in higher drug release amounts. With applied electrical voltages, the addition of PEDOT:PSS enhanced the drug release permeation due to the higher electrorepulsive forces from the cathode electrode and the PSS molecules, the cathode electrode and the BG chains, and the negatively charged PSS molecules and the BG chains.

In the previous works, Gamal et al. (2021) [26] investigated Sonidegib loaded ethosomes and liposomes to treat skin cancer. The amount of Sonidegib loaded in ethosomes was 10 mg. In the IN vitro drug releases (for 24 h), the amounts of Sonidegib released from ethosomes and liposomes were 61.87 ± 0.57% and 45.83 ± 0.62%, respectively. The corresponding amounts of Sonidegib release-permeation were 154.74 ± 1.72 μg/cm^2^ and 77.35 ± 0.89 μg/cm^2^, respectively. In the drug release kinetics, the Sonidegib release was suitably fitted to the Koremeyer-Peppas model (R^2^ = 0.9985) with the n value of 0.441 ± 0.007. In the in vivo study, the male mice (200–300 g) were used as the animal model and subcutaneously inoculated with 7,12-Dimethylbenz[a]anthracene (DMBA) to generate tumors. After 24 h, the mice were treated with Sonidegib-loaded liposome, showing a significant decrease in both hyperplasia (an increase in the number of cancer cells) and hyperkeratosis (an abnormal thickening of the stratum corneum). These suggested that they were nearly normal skins. This work also suggested that 10 mg of Sonidegib was an effective amount to treat skin cancer, as it could decrease the number of cancer cells and abnormal thickening of the stratum corneum. Pedersen et al. (2022) [27] studied Sonidegib release via topical delivery. In the preclinical studies, the porcine skin was treated once; the concentrations and times of Sonidegib release were 1–1.5 μg/g porcine skin and 1–8 h, respectively. The murine hair regrowth in the mouse model was treated once daily, in which hair growth was inhibited within 15 days. In the clinical study, the basal cell carcinoma patients (n = 61) were used as the human model, who were treated twice daily via topical delivery. The tumor volumes decreased by 53.4 ± 30.85% and 61.3 ± 31.18% after treatments of 4 and 9 weeks, respectively.

In our work, the amounts of imatinib loaded were 20 mg, whereas the amounts of Sonidegib permeated at 6 V were about 12.9 mg. The amount of 12.9 mg was well above the validated amount of 10 mg as shown in previous work. Thus, the present gelatin hydrogels were demonstrated here as an effective Sonidegib drug delivery matrix. In addition, the iontophoresis mode was effectively demonstrated in our work. This technique has not been previously reported for transdermal Sonidegib release.

## 4. Conclusions

PEDOT:PSS was synthesized as a drug carrier and successfully loaded with Sonidegib to enhance the Sonidegib release and release-permeation via the iontophoresis technique. BG hydrogel as the matrix was fabricated via physical crosslinking which was non-toxic towards human skin. The interactions between Sonidegib and PEDOT:PSS were investigated by FTIR, TGA, and XPS techniques. The release experiments were investigated under the effects of gelatin types, gelatin concentrations, electrical voltages, and PEDOT:PSS. For the effect of gelatin types, the BG hydrogel showed the highest amount of Sonidegib release compared to PG due to the higher electrorepulsive force. At higher concentrations, the amount of Sonidegib release decreased because of the higher entanglements and smaller mesh sizes. When PEDOT:PSS was loaded and acted as the drug carrier, the amount of Sonidegib release increased because PEDOT:PSS provided the highest electrorepulsive and electroosmosis forces. The release-permeation experiments (porcine skin as the membrane) were investigated under the effects of pH values, electrical voltages, and PEDOT:PSS. At a lower pH, the amount of Sonidegib release-permeation decreased due to a smaller electrorepulsive force. Under applied electrical voltages and added PEDOT:PSS, the amounts of Sonidegib release-permeation and diffusion coefficient increased, whereas the corresponding time to equilibrium decreased. The possible reasons were: PEDOT:PSS was incorporated into the BG matrix, resulting in the larger mesh sizes; PEDOT:PSS enhanced electrical response, leading to the higher electroosmosis and electrorepulsive forces. Sonidegib/PEDOT:PSS in the BG hydrogel has been demonstrated as a drug matrix system in which the Sonidegib release and release-permeation can be controlled effectively via the iontophoresis technique.

## Figures and Tables

**Table 1:** The degrees of swelling, weight losses, mesh sizes, and crosslinking densities of gelatin hydrogels of various types, concentrations, pH values, electric voltages, and with PEDOT:PSS.

Samples	pH Values	Electric Voltages (V)	% Swelling	% Weight Losses	Mesh Sizes (Å)	Crosslink Density (mol/cm^3^)
5% *v*/*v* BG	5.5	0	130.39 ± 9.36	52.51 ± 1.20	8.26 ± 2.5	1.5 × 10^−2^ ± 7.4 × 10^−3^
	7.4	0	336.40 ± 29.86	74.26 ± 1.03	16.4 ± 3.7	6.1 × 10^−3^ ± 1.8 × 10^−3^
		3	417.82 ± 39.94	75.55 ± 1.34	18.9 ± 4.1	3.8 × 10^−3^ ± 1.6 × 10^−3^
		6	544.42 ± 27.37	78.30 ± 0.45	28.5 ± 7.3	2.0 × 10^−3^ ± 9.2 × 10^−4^
PEDOT:PSS/5% *v*/*v* BG	7.4	0	384.73 ± 2.18	68.10 ± 0.96	19.9 ± 4.0	3.6 × 10^−3^ ± 1.1 × 10^−3^
		3	476.21 ± 22.05	78.14 ± 3.30	20.3 ± 1.8	3.1 × 10^−3^ ± 4.7 × 10^−4^
		6	976.10 ± 38.68	88.63 ± 0.22	23.5 ± 3.6	2.6 × 10^−3^ ± 6.1 × 10^−4^
6% *v*/*v* BG	7.4	0	73.02 ± 2.20	25.47 ± 2.82	10.7 ± 0.3	9.2 × 10^−3^ ± 4.7 × 10^−4^
7% *v*/*v* BG	7.4	0	26.59 ± 3.36	7.49 ± 0.73	5.4 ± 0.9	3.3 × 10^−2^ ± 9.7 × 10^−3^
5% *v*/*v* PG	7.4	0	111.39 ± 1.85	41.72 ± 0.44	9.7 ± 1.5	1.1 × 10^−2^ ± 2.5 × 10^−3^
6% *v*/*v* PG	7.4	0	31.41 ± 2.82	19.88 ± 3.20	4.6 ± 0.2	3.5 × 10^−2^ ± 7.8 × 10^−4^
7% *v*/*v* PG	7.4	0	6.90 ± 0.61	7.94 ± 2.65	3.4 ± 0.9	7.5 × 10^−2^ ± 1.7 × 10^−2^

**Table 2:** Kinetic factors and diffusion coefficients of Sonidegib released from the BG and PG gelatin hydrogels with a thickness of 2.0 ± 0.5 mm and an area of 3.14 cm^2^.

Conditions (Actual Drug)	Electric Voltages (V)	Power Law			Higuchi		D (cm^2^s^−1^)	C_0_ (gcm^−3^)	Amount of Sonidegib Released (mg)	% Release	Time to Equilibrium (h)
		n	k	r^2^	k_H_	r^2^					
5% *v*/*v* PG (19.5 ± 0.2)	-	0.34	2.55 × 10^−2^	0.87	5.30 × 10^−3^	0.94	2.82 × 10^−13^	21.23	7.62	38.10%	6.0
5% *v*/*v* BG (19.7 ± 0.1)	-	0.31	4.29 × 10^−2^	0.99	5.75 × 10^−3^	0.89	4.80 × 10^−13^	21.23	9.22	46.10%	3.5
5% *v*/*v* BG (19.7 ± 0.1)	3 (0.21 μA)	0.27	6.73 × 10^−2^	0.99	5.99 × 10^−3^	0.87	7.74 × 10^−13^	21.23	11.11	55.57%	3.0
5% *v*/*v* BG (19.7 ± 0.1)	6 (3.14 μA)	0.34	3.39 × 10^−2^	0.99	5.74 × 10^−3^	0.90	9.60 × 10^−13^	21.23	12.93	64.67%	2.5
6% *v*/*v* BG (19.3 ± 0.2)	-	0.46	1.02 × 10^−2^	0.96	5.54 × 10^−3^	0.96	2.77 × 10^−13^	21.23	7.24	36.22%	5.0
7% *v*/*v* BG (19.3 ± 0.3)	-	0.54	3.86 × 10^−3^	0.90	3.86 × 10^−3^	0.90	1.76 × 10^−13^	21.23	6.18	30.90%	7.0
PEDOT:PSS/5% *v*/*v* BG (19.6 ± 0.2)	-	0.52	6.28 × 10^−3^	0.93	5.52 × 10^−3^	0.92	7.56 × 10^−13^	21.23	11.94	59.68%	6.0
PEDOT:PSS/5% *v*/*v* BG (19.6 ± 0.2)	3 (1.66 μA)	0.42	1.57 × 10^−2^	0.99	5.65 × 10^−3^	0.91	9.53 × 10^−13^	21.23	13.15	65.77%	5.5
PEDOT:PSS/5% *v*/*v* BG (19.6 ± 0.2)	6 (3.11 μA)	0.65	1.82 × 10^−3^	0.89	5.46 × 10^−3^	0.93	1.05 × 10^−12^	21.23	14.26	71.29%	5.0

**Table 3:** Kinetic factors and diffusion coefficients of Sonidegib release-permeation from the 5% *v*/*v* BG and PEDOT:PSS/5% *v*/*v* BG hydrogels with a thickness of 2.0 ± 0.5 mm and an area of 3.14 cm^2^.

Conditions (Actual Drug)	pH Values	Electric Voltages (V)	Power Law			Higuchi		D (cm^2^s^−1^)	C_0_ (gcm^−3^)	Amount of Sonidegib Permeated (mg)	% Release	Time to Equilibrium (h)
			n	k	r^2^	k_H_	r^2^					
5% *v*/*v* BG (19.7 ± 0.1)	5.5	-	0.58	3.47 × 10^−3^	0.95	5.43 × 10^−3^	0.93	1.66 × 10^−13^	21.23	5.77	28.83%	6.0
5% *v*/*v* BG (19.7 ± 0.1)	7.4	-	0.55	4.53 × 10^−3^	0.97	5.44 × 10^−3^	0.94	2.34 × 10^−13^	21.23	6.76	33.81%	5.0
5% *v*/*v* BG (19.7 ± 0.1)	7.4	3 (0.12 μA)	0.64	1.80 × 10^−3^	0.95	5.36 × 10^−3^	0.93	3.81 × 10^−13^	21.23	8.78	43.90%	4.5
5% *v*/*v* BG (19.7 ± 0.1)	7.4	6 (2.13 μA)	0.46	1.03 × 10^−2^	0.99	5.50 × 10^−3^	0.92	5.42 × 10^−13^	21.23	10.16	50.78%	4.0
PEDOT:PSS/5% *v*/*v* BG (19.5 ± 0.2)	7.4	-	0.56	4.31 × 10^−3^	0.95	5.55 × 10^−3^	0.92	4.31 × 10^−3^	21.23	10.58	52.94%	6.0
PEDOT:PSS/5% *v*/*v* BG (19.5 ± 0.2)	7.4	3 (1.39 μA)	0.53	6.32 × 10^−3^	0.97	5.60 × 10^−3^	0.91	7.72 × 10^−13^	21.23	11.89	59.46%	5.0
PEDOT:PSS/5% *v*/*v* BG (19.5 ± 0.2)	7.4	6 (3.12 μA)	0.56	4.38 x10^−3^	0.97	5.50 × 10^−3^	0.93	8.59 × 10^−13^	21.23	12.88	64.38%	4.5

## Data Availability

The original contributions presented in this study are included in the article/Supplementary Materials. Further inquiries can be directed to the corresponding author.

## Supplementary Materials

The following supporting information can be downloaded at https://www.mdpi.com/article/10.3390/pharmaceutics18040494/s1, Characterizations; Table S1. FTIR data; Table S2. Thermal decomposition data; Table S3. T_d,onset,_ and % mass losses; Table S4. Elemental compositions from XPS survey scan spectra; Figure S1: Amounts of Sonidegib released from the BG gelatin hydrogels under the effect of BG concentrations. Figures in: (a) the Sonidegib amounts versus time in a log–log plot; and (b) the Sonidegib amounts versus time^1/2^. The dashed lines indicate the trend lines. Figure S2. Amounts of Sonidegib released from the BG and PG gelatin hydrogels under the effects of: (A) gelatin types (BG and PG); (B) electric fields (5% *v*/*v* BG); (C) PEDOT:PSS/5% *v*/*v* BG. The dashed lines indicate the trend lines. Figure S3. Amounts of Sonidegib released-permeated from the 5% *v*/*v* BG hydrogel through the pig skin under the effects of: (A) pH values; (B) electric fields; (C) PEDOT:PSS.
