# Supplementary material for: Transdermal Delivery of Sonidegib via Iontophoresis from PEDOT:PSS/Gelatin Hydrogels for Basal Cell Carcinoma Skin Cancer
Source: Pharmaceutics. 2026 Apr 17;18(4):494. doi: 10.3390/pharmaceutics18040494 (PMC13119114; doi:10.3390/pharmaceutics18040494)
Supplement: Supplementary file 1 [file pharmaceutics-18-00494-s001.zip › pharmaceutics-4213360-supplementary.pdf]

*Article*

# Transdermal Delivery of Sonidegib via Iontophoresis from PEDOT:PSS/Gelatin Hydrogels for Basal Cell Carcinoma Skin Cancer

**Phimchanok Sakunpongpitiporn [1], Johannes Schwank [2], Napa Parinyanitikul [3,4], Sutima Luangdilok [5], Nattaya Teeyapun [3,4], Aumpika Kesornsit [6], and Anuvat Sirivat [1,*]**

[1] The Petroleum and Petrochemical College, Chulalongkorn University, Bangkok 10330, Thailand; phimchanok.s18@gmail.com (P.S.); anuvat.s@chula.ac.th (A.S.)
[2] Department of Chemical Engineering, University of Michigan, Ann Arbor, MI 48109, USA; schwank@engin.umich.edu
[3] Division of Medical Oncology, Department of Medicine, Faculty of Medicine, Chulalongkorn University , Bangkok 10330, Thailand; napaparinyanitikul@gmail.com (N.P.); nattaya.te@chula.ac.th (N.T.)
[4] The King Chulalongkorn Memorial Hospital, Bangkok 10330, Thailand; napaparinyanitikul@gmail.com (N.P.); nattaya.te@chula.ac.th (N.T.)
[5] Department of Biochemistry, Faculty of Medicine, Chulalongkorn University, Bangkok 10330, Thailand; sutima.l@chula.ac.th
[6] Faculty of Pharmaceutical Sciences, Burapha University, Chonburi 20131, Thailand; aumpika.ke@go.buu.ac.th
* Correspondence: anuvat.s@chula.ac.th

## 1. Characterizations

A Fourier transform infrared spectrometer (FT-IR) (Nicolet, iS 5, ID 7, Bangkok,Thailand)) was used to investigate the functional groups and interactions of PEDOT:PSS, Sonidegib, Sonidegib/PEDOT:PSS, the gelatin hydrogels, Sonidegib/gelatin hydrogels, and Sonidegib/PEDOT:PSS/gelatin hydrogels. The instrument was operated in the absorbance mode, in the wavenumbers between 1000 and 3800 $cm^{-1}$, with 64 scans, a resolution of 4 $cm^{-1}$, and at room temperature.

A simultaneous thermal analyzer (STA) (Netzsch, STA 449F3, Perkin Elmer, Pyris Diamond, Waltham, MA, United States) was used to determine the thermal behaviors of PEDOT:PSS, Sonidegib, Sonidegib/PEDOT:PSS, the gelatin hydrogels, Sonidegib/gelatin hydrogels, Sonidegib/PEDOT:PSS/gelatin hydrogels. Temperature was varied between 30 and 800 °C at a heating rate of 10 °C $min^{-1}$ in a nitrogen atmosphere.

An X-ray photoelectron spectrometer (XPS) (Kratos, Axis Ultra DLD, Manchester, United Kingdom) was used to analyze the elements and interactions of PEDOT:PSS and Sonidegib/PEDOT:PSS. A monochromatic Al-Ka radiation source was employed at 160 eV for the survey scan and at 40 eV for the narrow scan. The C 1s spectrum was used as the reference. The Casa-XPS software was utilized in the data interpretation.

A scanning electron microscope (SEM) (Hitachi, S-4800, Tokyo, Japan) was used to investigate the morphology of gelatin hydrogels, PEDOT:PSS/gelatin hydrogels, PEDOT:PSS, and Sonidegib/PEDOT:PSS. It was operated at 2 kV and 10 mA current. In the sample preparation, the gelatin and PEDOT:PSS/gelatin hydrogels were immersed in a PBS buffer (pH 7.4) until reaching equilibrium. The samples were frozen in a refrigerator (Melling Biomedical, YCD-EL-300) at −20 °C for 7 days, and then were lyophilized in a freeze-drier (Biobase, BK-FD 10) at −60 °C for 16 hours. Finally, they were frozen with a liquid nitrogen (−40 °C).

**Table S1.** FTIR data.

| Samples | Wavenumber ($cm^{-1}$) | Meaning |
|---|---|---|
| 5%v/v BG hydrogel (Figure 2A(a)) | 3278 | the overlapping between the O-H and N-H stretching vibrations |
| | 1634 | C=O stretching of amide I (triple helices in BG hydrogel) |
| Sonidegib (Figure 2A(b)) | 3229 | N-H stretching vibration |
| | 1489−1397 | $CH_3$ stretching vibration |
| | 1373 | aromatic C-C vibration |
| | 1144 | C-H stretching vibration |
| PEDOT:PSS (Figure 2A(c)) | 3436 | O-H stretching vibration |
| | 1522 | C=C stretching vibration |
| | 1321 | C-C stretching vibration |
| | 1203 | S=O symmetric stretching vibration |
| | 1095 | S=O asymmetric starching vibration |
| Sonidegib/PEDOT:PSS (Figure 2A(d)) | 3276 | N-H stretching vibration (Sonidegib) |
| | 1531 | C=C stretching vibration (PEDOT:PSS) |
| Sonidegib/5%v/v BG hydrogel (Figure 2A(e)) | 3312 | the overlapping between the O-H and N-H stretching vibrations (BG hydrogel and Sonidegib) |
| | 1640 | C=O stretching of amide I (triple helices in BG hydrogel) |
| Sonidegib/PEDOT:PSS/ 5%v/v BG hydrogel (Figure 2A(f)) | 3271 | the overlapping between the O-H and N-H stretching vibrations (BG hydrogel and Sonidegib) |
| | 1645 | C=O stretching of amide I (triple helices in BG hydrogel) |

**Table S2.** Thermal decomposition data.

| Samples | Decomposition Temperatures (°C) | Meaning |
|---|---|---|
| 5%v/v BG hydrogel | 40−140 | the water evaporation |
| | 140−700 | the cleavage bonds of protein chains |
| Sonidegib | 180−500 | the decomposition of sonidegib |
| PEDOT:PSS | 40−140 | the water evaporation |
| | 140−300 | the decomposition of the PEDOT side chain |
| | 300−400 | the decomposition of PSS |
| | 400−700 | the decomposition of PEDOT |
| Sonidegib/PEDOT:PSS | 40−180 | the water evaporation |
| | 180−400 | the overlapping decompositions of the PEDOT side chain and Sonidegib |
| | 400−540 | the decomposition of PSS |
| | 540−700 | the decomposition of PEDOT |
| Sonidegib/5%v/v BG hydrogel | 40−140 | the water evaporation |

| | | |
|---|---|---|
| | 140−700 | the overlapping decompositions of the Sonidegib and protein chains |
| Sonidegib/PEDOT:PSS/ 5%v/v BG hydrogel | 40−140 | the water evaporation |
| | 140−700 | the overlapping decompositions of the PEDOT:PSS, Sonidegib, and protein chains |

**Table S3.** $T_{d,onset}$ and % mass losses.

| Samples | $T_{d,onset}$ (% Mass Losses) | | |
|---|---|---|---|
| | 1st decomposition stage | 2nd decomposition stage | 3rd decomposition stage |
| 5%v/v BG Hydrogel | 72 °C (90.99%) | 277 °C (10.08%) | - |
| Sonidegib | 347 °C (99.49%) | - | - |
| PEDOT:PSS | 221 °C (11.23%) | 315 °C (12.71%) | 456 °C (29.74%) |
| Sonidegib/PEDOT:PSS | 300 °C (46.54%) | 427 °C (17.69%) | 571 °C (29.33%) |
| Sonidegib/5%v/v BG Hydrogel | 71 °C (91.41%) | 260 °C (7.41%) | - |
| Sonidegib/PEDOT:PSS/5%v/v BG Hydrogels | 63 °C (92.42%) | 266 °C (5.94%) | |

**Table S4.** Elemental compositions from XPS survey scan spectra.

| Samples | O 1s | C 1s | N 1s | S 2p | F 1s | Na 1s |
|---|---|---|---|---|---|---|
| Sonidegib | 27.08 | 67.89 | 1.89 | - | 3.14 | - |
| PEDOT:PSS | 34.25 | 59.18 | - | 2.72 | - | 3.85 |
| Sonidegib/PEDOT:PSS | 28.46 | 69.29 | 1.32 | 0.54 | 0.38 | - |

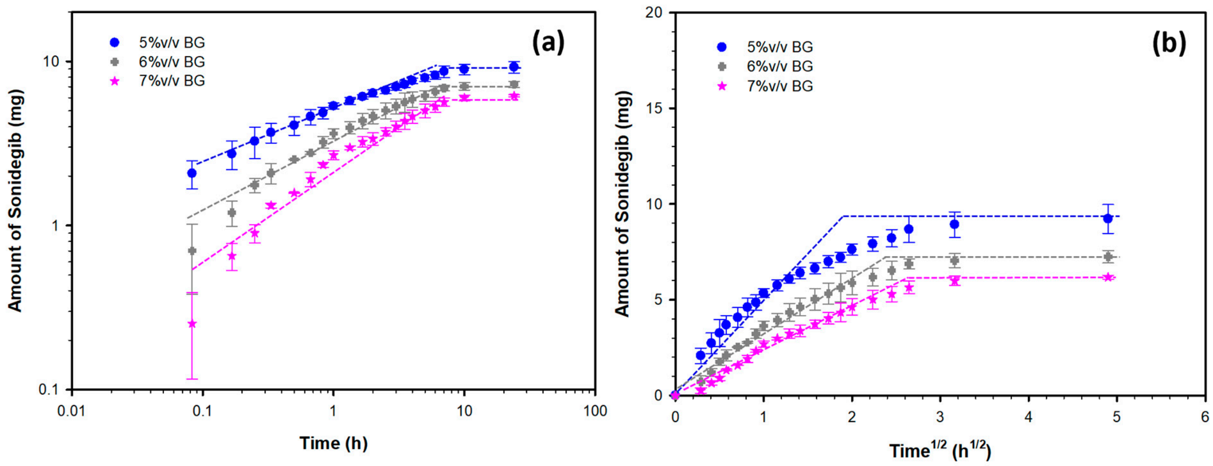


**Figure S1.** Amounts of Sonidegib released from the BG gelatin hydrogels under the effect of BG concentrations. Figures in: (a) the Sonidegib amounts versus time in a log–log plot; and (b) the Sonidegib amounts versus time$^{1/2}$. The dashed lines indicate the trend lines.

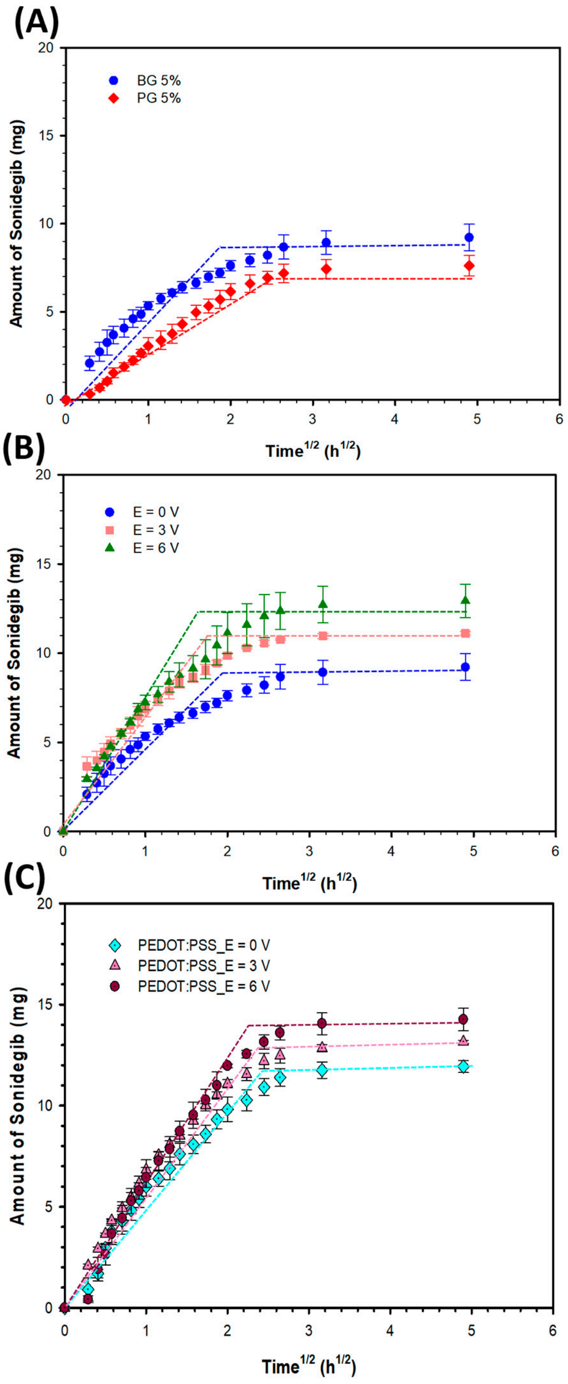


**Figure S2.** Amounts of Sonidegib released from the BG & PG gelatin hydrogels under the effects of**:** **(**A**)** gelatin types (BG & PG); **(**B**)** electric fields (5%v/v BG); **(**C**)** PEDOT:PSS/ 5%v/v BG**.** The dashed lines indicate the trend lines.

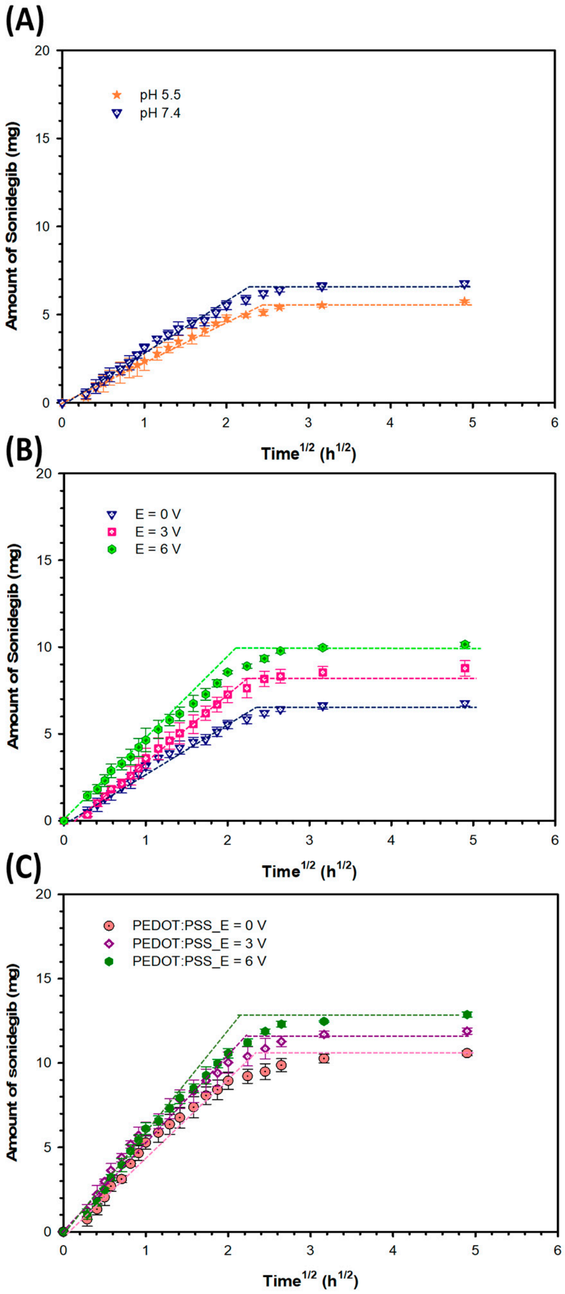


**Figure S3.** Amounts of Sonidegib released-permeated from the 5%v/v BG hydrogel through the pig skin under the effects of: (A) pH values; (B) electric fields; (C) PEDOT:PSS.
